# Role-play facilitates children’s mindreading of those with atypical color perception

**DOI:** 10.3389/fpsyg.2014.00817

**Published:** 2014-07-29

**Authors:** Fumikazu Furumi, Masuo Koyasu

**Affiliations:** ^1^Division of Cognitive Psychology in Education, Graduate School of Education, Kyoto University, KyotoJapan; ^2^Japan Society for the Promotion of Science, TokyoJapan

**Keywords:** cognitive development, mindreading, role-play, theory of mind, color perception, second-order false belief task

## Abstract

The present study examined the effects of role-play experience on children’s mindreading ability. Forty-one primary school children (20 boys, 21 girls, mean age: 9.37 years, range: 8–11 years) were introduced to a communication task in which the use of mindreading was essential. During each trial, participants viewed a shelf, presented on a laptop, which contained several familiar objects, and they were instructed to touch an object on the shelf following an order issued by a “manager” who stood at the opposite side of the shelf. There were two managers: one was a monkey manager with normal color vision, and the other was a dog manager with restricted color vision. The monkey manager could see all the objects in the same colors as the participants, whereas the dog manager saw some objects in different colors. Participants were required to respond according to the manager’s instruction. In the restricted color vision condition, the dog manager saw the colors of objects differently; thus, participants had to work out his intentions, according to his different perspective. In the normal color vision condition, all objects were in the same colors as those seen by the monkey manager. Before the test phase, participants in the role-play group were provided a role-play experience in which they assumed the role of the dog manager with restricted color vision. The experimental data were analyzed using a 2 × 2 mixed-design ANOVA (role-play condition × communication partner condition) to examine differences in the error rate. Both main effects and its interaction were significant. According to the *post-hoc* analyses, participants in the no-role-play condition made significantly more errors in the restricted color vision condition than in the normal color vision condition, whereas no such difference was found among participants in the role-play condition. These results suggest that role-play experience could facilitate mindreading of characters with restricted color vision.

## INTRODUCTION

Many previous studies have used false belief tasks to measure children’s mindreading ability (e.g., Wimmer and Perner, 1983; Baron-Cohen et al., 1985; Perner et al., 1989). For example, in the commonly used Sally-Anne task (Baron-Cohen et al., 1985), Sally puts her ball in a basket and she goes away. Then, Anne moves the ball to a box in Sally’s absence. Finally, Sally comes back to the scene. Children are asked where Sally will look for her ball. When children solve such tasks, they think implicitly that the characters have the same perceptual abilities and characteristics as they do. Because the Sally Anne task is too easy for typically developing adults, other tasks have been developed and used for measuring adults’ mindreading. Recent studies have shown, however, that it is difficult even for adults to interpret the mental states of others who have different characteristics (Komeda et al., 2009, 2013). Komeda et al. (2009) suggested that people can easily understand others’ minds when they read the minds of those who are similar to themselves. In their study, participants read a story and rated the protagonist’s emotional states. The results showed that extraverted participants rated correctly when the protagonist was extraverted. Komeda et al. (2013) also found that autistic people can read and memorize stories with an autistic protagonist more easily than that with a typically developing protagonist. On the other hand, typically developing people can read and memorize the story with a typically developing protagonist more easily than that with autistic protagonist. These studies suggest that people have difficulty in understanding others with different characteristics. Thus, mindreading ability of those with different characteristics is also important in child development, but it cannot be assessed through the use of traditional false belief tasks.

Over the last three decades, high-level mindreading has attracted researchers’ attention. High-level mindreading is a flexible system that develops throughout childhood and adolescence (Dumontheil et al., 2010a; Apperly, 2011). Apperly (2011) put forward two systems of mindreading. He suggested that social experience is needed for high-level mindreading development. One of the most important social experiences in childhood is role-play. Recent studies have revealed a positive role-play effect for adults and children. Goldstein and Winner (2012) found that children (aged 7–10 years) who were enrolled in after-school acting classes were better at an empathy task than same-age children who were enrolled in after-school visual arts classes. In addition, adolescents (aged 13–16 years) in acting classes were found to be better at an empathy task and a theory of mind task than those who were in other arts classes. Furumi and Koyasu (2012) explored the effect of social experience on mindreading development by focusing on role-play experience. The study showed that role-play has a positive effect on mindreading. The Director Task (Keysar et al., 2000; Dumontheil et al., 2010a) was used in this study. The Director Task is useful for investigating online mindreading skills for a wide age range of participants. In the Director Task, participants have to respond according to a director’s instruction. The director tells the participants to take an object from a shelf; however, the shelf has 4 closed slots out of16, so some of the objects cannot be seen from the director’s viewpoint, whereas participants can see all of the objects. In Furumi and Koyasu (2012), participants were assigned to one of two groups: role-play and no-role-play. The role-play group played the director’s role before the task. In contrast, the no-role-play group only watched another’s role-play on a screen. The role-play group made fewer errors than did the no-role-play group. Furumi (2013) also conducted the same study for children aged 8–11 years, and the same pattern of results was found. The study also found that children who passed all of the tasks of understanding others’ minds (second-order false belief task, Perner and Wimmer, 1985; distinguishing irony from deception, Winner and Leekam, 1991; commitment task, Mant and Perner, 1988) responded more accurately. Moreover, the role-play effect was different between the children who passed all of the tasks of understanding others’ minds (high score group) and those who did not (low score group). The role-play was found to be more effective for the low score group than for the high score group.

In this study, we focused on atypical color perception as an underlying cause of a different characteristic. To control color perception is easy and suitable for the Director Task. Furumi and Koyasu (2013) have shown that reading the mind of people with a different color perception is more difficult for adults than that of reading the mind of people with the same color perception, through the use of a modified version of the Director Task. In the task, there were two directors: monkey with a typical color vision, and Dog with an atypical color perception. Monkey could see all of the objects in the same colors as the participants, whereas Dog saw some objects in different colors (e.g., he saw as yellow objects that the participants saw as red). Participants were required to respond according to the directors’ instruction. In the atypical color perception condition, Dog saw the colors of objects differently; thus, participants had to work out his intentions (i.e., mindread), according to his different perspective. In the typical color vision condition, all objects were in the same colors as those seen by Monkey. Participants had to respond according to the directors’ intention based on their own perceptions. So, if Dog said “yellow,” he was referring to yellow from his own perspective, not from that of the participants’. Before the test phase, participants in a role-play group enacted the role of a person with atypical color perception. On the other hand, participants in a no-role-play group only watched another’s role-play. The results revealed that the no-role-play group made more errors than the role-play group.

In the present study, we modified the color version of the Director Task for children to investigate the effect of role-play provision in children. In the task we used, participants had to read the minds of others without using their own experience as a cue. We also used a second-order false belief task to confirm the relationship between the new mindreading task and a traditional false belief task.

We set up two hypotheses. Hypothesis A was that people who experience role-play as an atypical color perception director would be better at reading the minds of characters with atypical color perception, whereas people who do not experience this role-play would have difficulty reading the minds of characters with atypical color perception. Hypothesis B was that when a director has atypical color perception, children who fail a second-order false belief task would make more errors than children who pass it.

## MATERIALS AND METHODS

### PARTICIPANTS

A total of 41 children (mean age = 9.37 years, range = 8–11 years, 20 boys and 21 girls) were randomly assigned to either a role-play group (mean age = 9.23, range = 8–11, 9 boys and 12 girls) or a no-role-play group (mean age = 9.50, range = 8–11, 11 boys and 9 girls). Five additional children were tested but not included in the final sample because of technical difficulties (two girls) and performance in the Color Mate Test (three boys; explained in the next paragraph). The children were recruited from the cities of Kobe and Kyoto in Japan. Although some detailed demographic characteristics of the children could not be obtained because of a privacy policy that applied to data collection, they were predominantly from middle-class families. All spoke Japanese as their first language.

### Materials

#### Color mate test

**FIGURE 1:**
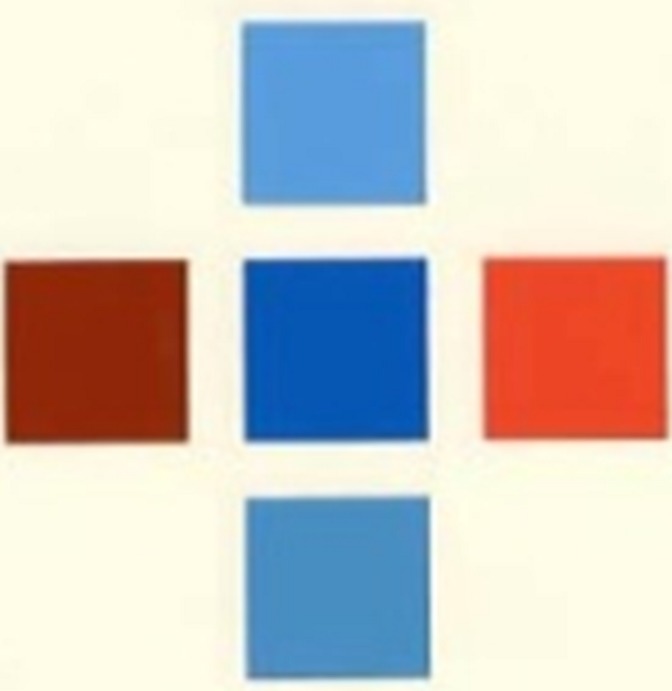
**Example of the Color Mate Test (Kaneko, 1995).** Participants were asked which line is a similar color: vertical or horizontal. In the case shown, vertical line consists of three blue colors. (http://nodaiweb.university.jp/cms/)

The Color Mate Test (Kaneko, 1995) is a simple test for determining how people perceive colors. Five different color squares make the shape of a cross on each piece of paper (see **Figure 1**). Participants were asked which line was of a similar color: vertical or horizontal. One practice trial and four test trials were presented. We used this test to see whether the participants had typical color vision. Participants who answered atypically in more than two trials (three boys) were omitted from the data analysis because they might have experienced difficulties in judging the colors presented in the color version of the Director Task.

#### Second-order false belief task

We used a second-order false belief task (e.g., Perner and Wimmer, 1985; Astington et al., 2002) which we presented with the use of animation on a laptop computer. The animation consisted of a main story, a belief question, a reality question, and a memory question as described below:

Main story: this is a dog’s house. The dog took a drum from the box and played with it. The dog put the drum into the box and went out. A cat came to play. The cat took the drum from the box and played. The cat put the drum into the bag. The dog saw this from the window, but the cat did not notice him watching. The dog came back into the room to play with the drum. Belief question: where does the cat think the dog will look for the drum?

Reality question: where is the drum now?

Memory question: where did the dog put the drum?

Children who answered all of the three questions correctly passed this task. We split the participants into two groups: those who passed this second-order false belief task and those who failed.

#### Director task

**FIGURE 2:**
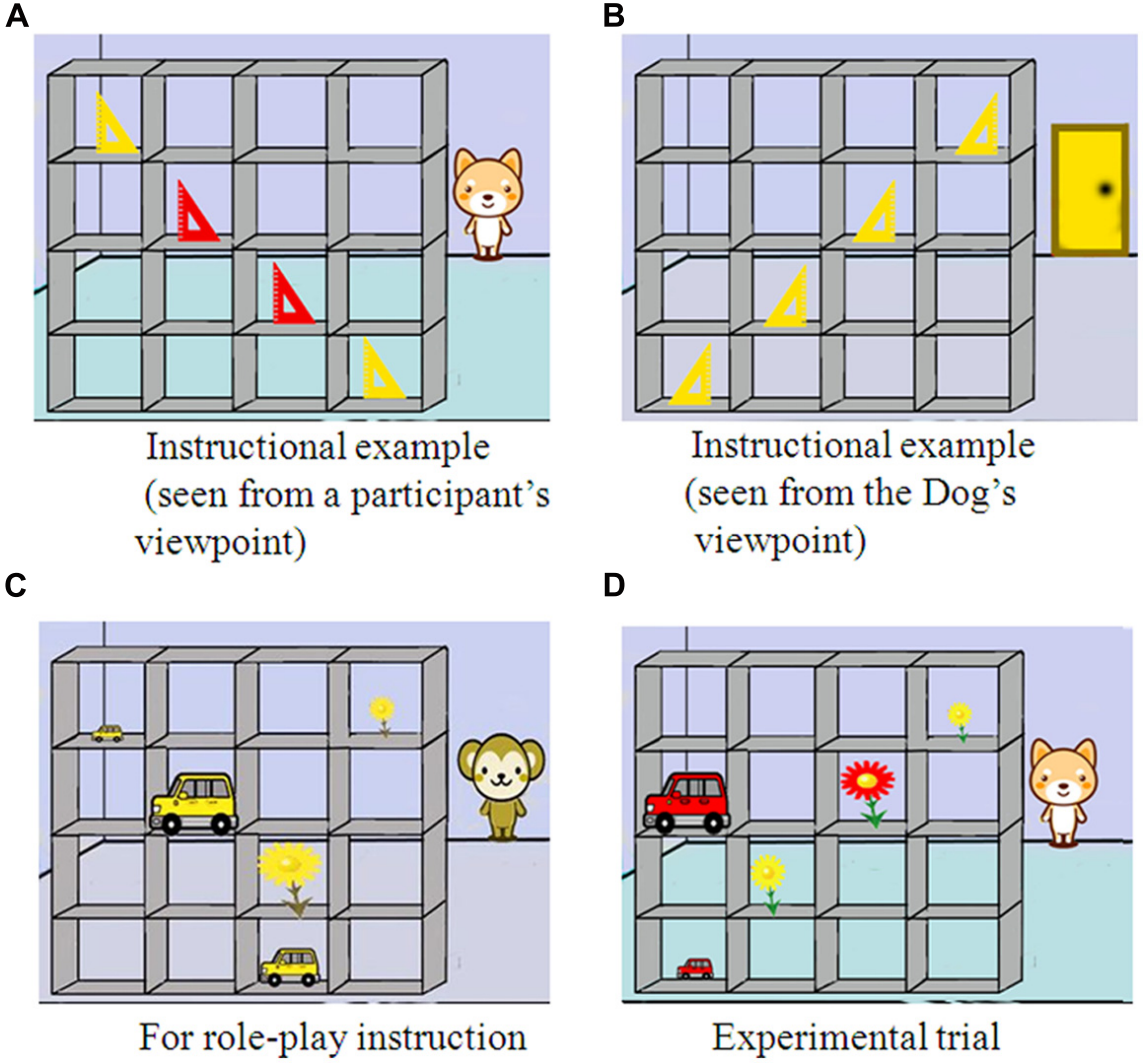
**Examples of stimuli used in the study.** **(A)** shows instructional example (seen from a participant’s viewpoint). **(B)** shows instructional example (seen from the Dog’s viewpoint). **(C)** shows an example stimulus for role-play instruction. **(D)** shows an example stimulus for experimental trial.

We used two laptops for the Director Task. We used a regular laptop (Sony VAIO VPCEA1AFJ) for instructions and another laptop with a touch screen (Fujitsu LifeBook AH/R3) for practice trials and test trials. We made and edited picture stimuli using Adobe Photoshop, and recorded verbal stimuli with an IC recorder (Sony ICD-SX850). We used Microsoft PowerPoint 2007 to make the materials for the role-play and no-role-play instructions. The stimuli for the practice and test trials were made and presented using SuperLab 4.0. We used 210 × 297 cm pieces of paper as order sheets for the role-play instructions. Examples of the picture stimuli are shown in **Figure 2**.

### PROCEDURE

First, we checked the color perception of all participants using the Color Mate Test. Then, we presented the second-order false belief task. After these tasks, all participants were introduced to the Director Task. Procedures used in the administration of the Director Task were as follows:

#### First instruction for both groups

Standardized instructions were presented on one of the laptop computers and read aloud by the experimenter. The Director Task was introduced as a “Shop Game” to participants. The players were the participant, Monkey, and Dog. There were a manager’s role and a clerk’s role. The manager told the clerk to choose an object from a shelf with 4 × 4 slots. Monkey had typical color vision, whereas Dog had atypical color perception. This information was explained using examples which were repeated a number of times. For example, Dog saw as yellow objects what the participants saw as red and yellow objects (see **Figures 2A,B**). Initially, participants had to answer a color question to confirm that they had typical color vision. After the instructions, those assigned to the role-play group had a role-play experience (the atypical color perception experience), whereas those assigned to the no-role-play group only watched another’s role-play situation.

#### Instruction for role-play group

The role-play group enacted the role of Dog manager with atypical color perception. Monkey played the clerk’s role. The participants told Monkey to take objects according to the order sheets with instructions written as “Can I have the “size (big or small)” yellow “object’s name (flower or car)”?” Five order sheets were given to the participants. In the role-play instructions, atypical color perception pictures (see **Figure 2C**) were presented for participants to experience atypical color perception. In line with procedures used in a previous study (cf. Furumi and Koyasu, 2013), Monkey responded correctly only to the third order. Monkey, with typical color vision, made four mistakes because it was assumed that the participants had atypical color perception. The experimenter asked participants whether Monkey took the right objects. All the participants answered “no” and gave the correct reason why Monkey chose the wrong objects. For example: “because Monkey has a different perception of colors from me.”

#### Instruction for no-role-play group

The experimenter presented the same picture stimuli and animations as in the role-play instructions provided to participants; however, participants only watched another’s role-play. Participants saw Dog’s restricted color world, and there was no difference in the visual experience between the role-play and no-role-play groups. The information included in these instructions matched the role-play instructions because the experimenter provided the same instructions to participants as in the role-play group. The only difference between the groups in these instructions was whether the participants experienced the role and communicated with Monkey. The experimenter asked participants whether Monkey took the right objects in the role-play session they observed. All of the participants answered “no” and gave the correct reason why Monkey chose the wrong the objects. For example: “because Monkey has a different perception of colors from Dog.”

#### Second instruction for both groups

The experimenter told the participants that they would play the clerk’s role and that Monkey and Dog would play the manager’s role. The experimenter repeated that Dog had atypical color perception, whereas Monkey had typical color vision. Moreover, the experimenter emphasized that the participants were required to respond according to the manager’s instruction: dog manager saw the colors of objects differently; thus, participants had to work out his intentions (i.e., mindread), according to his different perspective, whereas all objects were in the same colors as those seen by Monkey manager.

#### Practice trials for both groups

Before the test trials, participants took part in 10 practice trials to get used to the touch screen. The picture stimuli were presented, which included a patrol car and an airplane on a shelf with 4 × 4 slots, along with a recording of a woman’s voice saying “a patrol car” or “an airplane.” The participants touched the object on the screen according to the voice stimuli. The next trial was presented only after participants had responded correctly to the previous trial. When the participants finished the fifth trial, there was an interval during which the experimenter provided some feedback to participants about their touch responses. All participants became accustomed to the touch responses during the practice trials, as indicated by their satisfactory completion of those trials.

#### Test trials for both groups

The test trials consisted of two conditions: atypical color perception and typical color vision. Each condition included 20 trials. We adopted a block design and did not mix the two conditions within a block. Each block consisted of 10 trials. The blocks alternated between typical color vision condition and atypical color perception condition (e.g., 10 trials of typical color vision, 10 trials of atypical color perception, 10 trials of typical color vision, and 10 trials of atypical color perception). The condition that was presented in the first block was counterbalanced across participants. When the block condition changed, instructions about the next trial were shown on the laptop screen (e.g., Dog is the manager in the next block). In the atypical color perception condition, Dog was the director. On the other hand, in the typical color vision condition, Monkey was the director. The picture stimuli for the test trials were as follows: the director (Dog or Monkey) stood at the opposite side of five objects on a shelf with 4 × 4 slots. All the groups of objects consisted of two yellow objects and three red objects (see **Figure 2**). A woman’s voice (which was different from that used in the practice trials) was used for Monkey’s voice and a man’s voice was used for Dog’s voice. The participants had to touch the appropriate object according to the director’s instruction. The instructions from Dog and Monkey included size (big or small), color (yellow or red), and the object’s name (car or flower). For example, when the picture on **Figure 2D** was presented and Dog said “Can I have the big yellow flower?” participants should touch the biggest flower (red one). In the atypical color perception condition, 4 trials did not require mindreading of a character with atypical color perception because there was no discrepancy between the perspectives of Dog and participants. For example, when the picture on **Figure2D** was presented and Dog said “Can I have a small yellow flower?” participants should touch the smaller yellow flower. Trials that did not require mindreading were only used to confirm that they were seriously undertaking the experimental task. Only responses from 16 trials from the atypical color perception condition and 16 trials from the typical color vision condition were included in the final analyses.

#### Interview for both groups

After the test trials, the experimenter asked the participants questions, including what strategies they used. For example, “When Dog said …, how did you decide your response?” This interview was to check whether participants used a strategy that did not relate to mindreading; however, none of the participants used such a strategy.

## RESULTS

Twenty-three children (mean age = 9.48 years, range = 8–11 years, 11 boys and 12 girls) passed the second-order false belief task and 18 children (mean age = 9.22 years, range = 8–11 years, nine boys and nine girls) failed. This result is consistent with previous studies using the second-order false belief task (cf. Koyasu et al., 1998). We split the children into two groups according to whether they passed the second-order false belief task. There were 16 critical atypical color perception condition trials and 16 corresponding trials in the typical color vision condition. A division of the number of correct answers by 16 (the number of critical trials) was used for calculating each accuracy rate (%). The median reaction times (ms) were calculated from correct responses for each child. First, we checked the order effect. There was no significant difference between the two groups of experimental orders both for accuracy data [typical color vision condition: *t*(39) = 0.82, *p* = 0.07, *r* = 0.13; atypical color perception condition: *t*(39) = 0.31, *p* = 0.41, *r* = 0.05] and for reaction time data [typical color vision condition: *t*(39) = 0.09, *p* = 0.61, *r* = 0.01; atypical color perception condition: *t*(39) = 0.36, *p* = 0.52, *r* = 0.06]. Second, we checked the gender effect. There was no significant difference between genders both for accuracy data [typical color vision condition: *t*(39) = 0.51, *p* = 0.62, *r* = 0.08; atypical color perception condition: *t*(39) = 0.62, *p* = 0.54, *r* = 0.10] and for reaction time data [typical color vision condition: *t*(39) = 0.77, *p* = 0.07, *r* = 0.12; atypical color vision condition: *t*(39) = 0.49, *p* = 0.63, *r* = 0.08]. Third, we conducted 2 (role-play: role-play or no-role-play) × 2 (director: restricted color or normal color) × 2 (second-order false belief: pass or fail) analyses of covariance (ANCOVAs), with the two between-participants factors (role-play and second-order false belief) and one within-participants factor (director) to examine differences in the accuracy rate and reaction times for atypical color perception condition and typical color vision condition. Age was included in the analyses as covariates to control for the age effect.

### ACCURACY DATA

**FIGURE 3:**
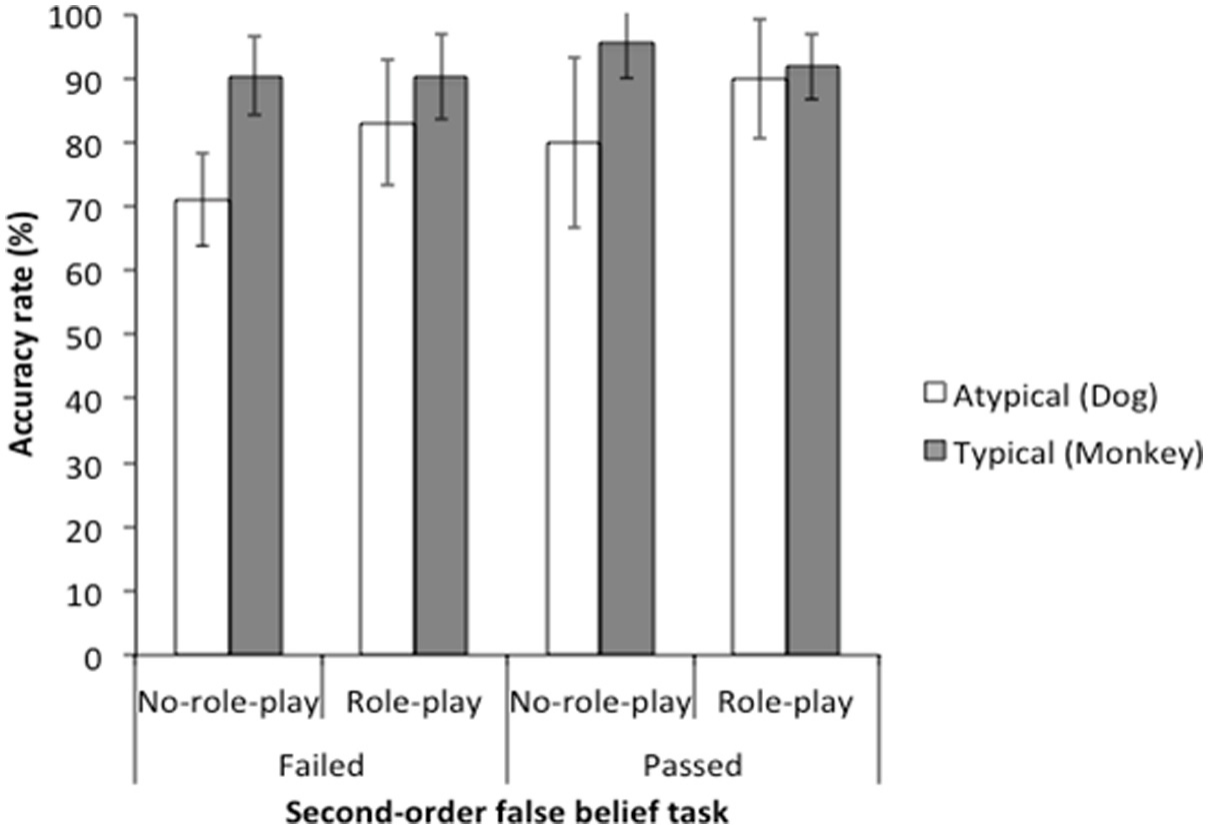
**Means (and Standard Deviations) of accuracy rates**.

We verified that age did not affect the accuracy rates [*F*(1,36) = 1.47, *p* = 0.23, $\eta_{p}^{2}$ = 0.04]. Then, we conducted 2 (role-play: role-play or no-role-play) × 2 (director: restricted color or normal color) × 2 (second-order false belief: pass or fail) mixed-design ANOVA (analysis of variance). All main effects were significant [role-play: *F*(1,37) = 5.00, *p* = 0.032, $\eta_{p}^{2}$ = 0.12; director: *F*(1,37) = 33.08, *p* < 0.001, $\eta_{p}^{2}$ = 0.47; second-order false belief: *F*(1,37) = 8.04, *p* = 0.007, $\eta_{p}^{2}$ = 0.18]. Role-play group (*M* = 87.50, SD = 10.34) responded more accurately than no-role-play group (*M* = 75.30, SD = 11.77). Children responded more accurately in the typical color vision condition (*M* = 92.07, SD = 6.25) than in the restricted color vision condition (*M* = 81.25, SD = 12.58). Children who passed the second-order false belief task (*M* = 85.60, SD = 12.56) responded more accurately than those who failed it (*M* = 75.69, SD = 10.48). Role-play × director interaction [*F*(1,37) = 11.44, *p* = 0.002, $\eta_{p}^{2}$ = 0.24] was also significant. According to *post-hoc* analyses we conducted, no-role-play participants responded more accurately in the typical color vision condition (*M* = 92.86, SD = 6.64) than in the atypical color perception condition [*M* = 75.30, SD = 11.77; *t*(20) = 6.92, *p* < 0.001 (two-tailed), *r* = 0.84], whereas there was no difference between the conditions (typical color vision condition, *M* = 91.25, SD = 5.88; atypical color perception condition, *M* = 87.50, SD = 10.34) among role-play participants [*t*(19) = 1.39, *p* = 0.18 (two-tailed), *r* = 0.30]. Other interactions were not significant [role-play × director × second-order false belief: *F*(1,37) = 0.04, *p* = 0.84, $\eta_{p}^{2}$ = 0.001; role-play × second-order false belief: *F*(1,37) = 0.51, *p* = 0.48, $\eta_{p}^{2}$ = 0.01; director × second-order false belief: *F*(1,37) = 1.36, *p* = 0.25, $\eta_{p}^{2}$ = 0.04] **Figure 3** shows the means and SDs of accuracy rates.

### REACTION TIME DATA

**FIGURE 4:**
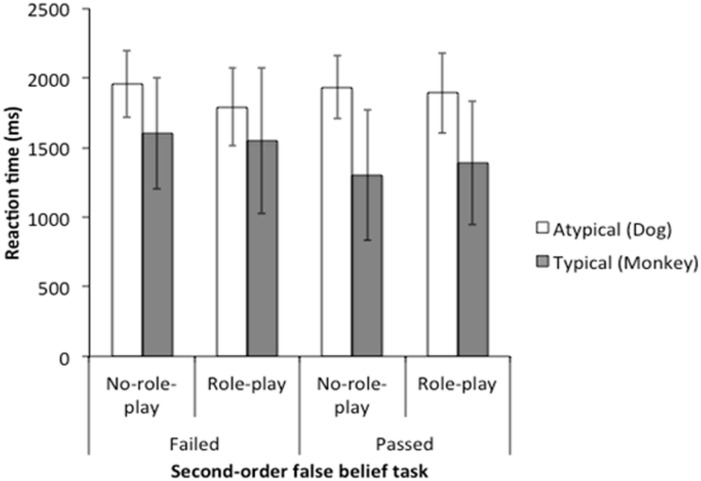
**Means (and Standard Deviations) of reaction times**.

We verified that age did not affect the reaction times [*F*(1,36) = 2.35, *p* = 0.13, $\eta_{p}^{2}$ = 0.06]. Then, we conducted 2 (role-play: role-play or no-role-play) × 2 (director: restricted color or normal color) × 2 (second-order false belief: pass or fail) mixed-design ANOVA. Director main effect was significant [*F*(1,37) = 44.93, *p* < 0.001, $\eta_{p}^{2}$ = 0.55]. Children responded more rapidly in the typical color vision condition (*M* = 1451.15, SD = 473.87) than in the atypical color perception condition (*M* = 1901.61, SD = 269.97). Director × second-order false belief interaction [*F*(1,37) = 4.26, *p* = 0.046, $\eta_{p}^{2}$ = 0.10] was also significant. According to *post-hoc* analyses we conducted, children who passed the second-order false belief task responded more rapidly in the typical color vision condition (*M* = 1350.83, SD = 466.01) than in the atypical color perception condition [*M* = 1909.26, SD = 269.79; *t*(22) = 6.13, *p* < 0.001 (two-tailed), *r* = 0.80]. Children who failed the second-order false belief task also responded more rapidly in the typical color vision condition (*M* = 1579.33, SD = 465.08) than in the atypical color perception condition [*M* = 1891.83, SD = 277.69; *t*(17) = 3.91, *p* = 0.001 (two-tailed), *r* = 0.69]. There was no difference between children who passed the second-order false belief task (*M* = 1350.83, SD = 466.01) and children who failed the second-order false belief task (*M* = 1579.33, SD = 465.08) for reaction time in typical color vision condition [*t*(39) = 1.56, *p* = 0.13 (two-tailed), *r* = 0.24]. There was no difference between children who passed the second-order false belief task (*M* = 1909.26, SD = 269.787) and children who failed the second-order false belief task (*M* = 1891.83, SD = 277.69) for reaction time in atypical color vision condition [*t*(39) = 0.20, *p* = 0.84 (two-tailed), *r* = 0.03]. Other main effects and interactions were not significant [role-play: *F*(1,37) = 0.16, *p* = 0.69, $\eta_{p}^{2}$ = 0.004; second-order false belief: *F*(1,37) = 0.80, *p* = 0.38, $\eta_{p}^{2}$ = 0.02; role-play × director: *F*(1,37) = 0.84, *p* = 0.37, $\eta_{p}^{2}$ = 0.02; role-play × second-order false belief: *F*(1,37) = 0.37, *p* = 0.55, $\eta_{p}^{2}$ = 0.01; role-play × director × second-order false belief: *F*(1,37) = 0.001, *p* = 0.98, $\eta_{p}^{2}$ < 0.001]. **Figure 4** shows the means and SDs of reaction times.

## DISCUSSION

In this study, we investigated the ability of children to read the minds of characters with atypical color perception. Our color version of the Director Task can measure participants’ ability to read the minds of characters with different characteristics, which cannot be identified by traditional mindreading tasks. Participants had to inhibit their own color perception and to infer others’ instructions on critical trials. Before the test trials, we confirmed the children’s understanding of the situation: monkey had the same color perception as that of the participants and Dog had a different color perception. According to the reaction time data, however, children spent more time in the restricted color condition than typical color vision condition, which means reading the mind of individuals with atypical color perception requires more time for children with typical color vision in online communication. During the test trials, no-role-play group children made more errors when the director had atypical color perception This finding supports hypothesis A: people who experience the role of an atypical color perception director can read the minds of characters with atypical color perception, whereas people who do not experience the role of an atypical color perception director have difficulty reading the minds of characters with atypical color perception. There was no difference among participants when the director had typical color vision. This means that there was no difference about the ability to solve the Director Task between the groups. These are consistent with the previous research results on adults (e.g., Furumi and Koyasu, 2013). It is important, however, that children’s accuracy rates were not much lower than those of the adults’ accuracy rates in the previous research (accuracy rates were about 73% for no-role-play group and about 95% for role-play group, Furumi and Koyasu, 2013), which contrasts with the findings of previous studies using this task with children and adults (cf. Dumontheil et al., 2010a; Furumi, 2013). The present results suggest that the development of mindreading does not necessarily follow a linear trajectory and highlight the importance of research into this socio-cognitive domain for future studies.

We also found that this color perception version of the Director Task relates to the traditional second-order false belief task (Perner and Wimmer, 1985). In the atypical color perception condition, children who failed the second-order false belief task made more errors than those who passed it. On the other hand, in the typical color vision condition, there was no difference among the children. This result supports hypothesis B: when a director has atypical color perception, children who fail a second-order false belief task would make more errors than children who pass it. On the other hand, when a director has typical color vision, there is no difference between the two groups. This finding suggests that understanding the minds of others with different characteristic requires high level mindreading skill.

No significant interaction was found between role-play and second-order false belief. Furumi (2013) found that role-play has a stronger effect on children with a low ability to mindread than on children with high mindreading ability. The results of this study are not consistent with those of previous research in terms of the difference of the role-play effect. Furumi (2013) used the occlusion version of the Director Task (cf. Dumontheil et al., 2010a). In the occlusion version Director Task, participants should consider which object can be seen by the director. Dumontheil et al. (2010b) suggested that such a Director Task involves level 1 perspective taking (understanding that people with different lines of sight might see different things when there is a wall between them: Flavell et al., 1981; Apperly, 2011). On the other hand, Furumi and Koyasu (2013) suggested that the color version Director Task involves level 2 perspective taking (understanding that people with different lines of sight might see the very same things but in different ways: Flavell et al., 1981; Apperly, 2011). The different level of perspective taking might have caused the different findings relating to the role-play effect. We have not identified yet what is the most important factor in the color version Director Task. We aim to elucidate this factor and related issues in future research.

Most previous mindreading tasks have been created by incorporating discrepancies in the situations presented to participants. For example, in the Sally-Anne task (Baron-Cohen et al., 1985), the different mental states between Sally and participants are not attributed to Sally herself, but attributed to the situation (Sally did not see that Anne moved the ball). In contrast, the color version Director Task used in the present study creates discrepancy by presenting personal differences (Monkey has typical color vision and Dog has atypical color perception). Additionally, we have also found that role-play has positive effects on reading the mind of characters with different perception. Recent studies have described methods of training mindreading ability for adults (Furumi and Koyasu, 2012, 2013; Santiesteban et al., 2012). The present study has revealed that role-play experience is also effective for enhancing children’s mindreading skills.

We have found that role-play helps children to read the mind of character with atypical color vision. However, it is still unclear what the mechanism of role-play is. One of our future plans is to confirm the effect of role-play. In the present study, two characters, Dog and Monkey, appeared in the Director Task. All the role-play group participants played Dog’s role, but not Monkey’s role. In future research, establishing a new control group playing Monkey’s role will help to confirm some aspects of the role-play mechanism. Another future plan is to investigate how long the effect of role-play would last. In the present study, all the role-play group participants did the Director Task just after the role-play session. To confirm how long the effect lasts would help to clarify aspects of the role-play mechanism and the associated benefits for children.

In conclusion, the findings of this study indicate that role-play has a positive effect on children’s ability to read the mind of others with different perceptions. This suggests that role-play could be used as an effective intervention for children who have difficulty in mindreading. Revealing the mechanisms of role-play in further studies is important to develop training and interventions to improve mindreading skills. Furthermore, this new type of mindreading task might measure different aspects of mindreading compared to traditional mindreading tasks. Many researchers have pointed out various kinds of mindreading, such as spontaneous mindreading (Senju, 2012), high-level and low-level mindreading (Apperly, 2011), as well as implicit and explicit mindreading (Low and Perner, 2012). We could add “reading the mind of characters with different perception from us” to the list. It is important to find out developmental trajectories of these various types of mindreading and to know the whole structure of mindreading development in future studies.

## Conflict of Interest Statement

The authors declare that the research was conducted in the absence of any commercial or financial relationships that could be construed as a potential conflict of interest.
